# *Bulnesia sarmientoi* Supercritical Fluid Extract Exhibits Necroptotic Effects and Anti-Metastatic Activity on Lung Cancer Cells

**DOI:** 10.3390/molecules23123304

**Published:** 2018-12-13

**Authors:** Heng-Long Wang, Jung-Che Chang, Li-Wen Fang, Hsia-Fen Hsu, Li-Chiun Lee, Jyh-Ferng Yang, Ming-Tsai Liang, Pei-Chi Hsiao, Chao-Ping Wang, Shih-Wei Wang, Chi-Chang Chang, Jer-Yiing Houng

**Affiliations:** 1 Department of Life Sciences, National University of Kaohsiung, Kaohsiung 81148, Taiwan; hlwang@nuk.edu.tw (H.-L.W.); jungche2004@yahoo.com.tw (J.-C.C.); 2 Department of Nutrition, I-Shou University, Kaohsiung 82445, Taiwan; fanglw@isu.edu.tw (L.-W.F.); fen153848@gmail.com (H.-F.H.); edman@isu.edu.tw (L.-C.L.); 3 Graduate Institute of Biotechnology and Chemical Engineering, I-Shou University, Kaohsiung 84001, Taiwan; jfyang@isu.edu.tw (J.-F.Y.); mtliang@isu.edu.tw (M.-T.L.); 4 Metal Industries Research & Development Centre, Kaohsiung 81160, Taiwan; peg3319@gmail.com; 5 Divisions of Cardiology, E-Da Hospital/I-Shou University, Kaohsiung 82445, Taiwan; ed100232@edah.org.tw; 6 Division of Allergy, Immunology, and Rheumatology, Department of Internal Medicine, E-Da Hospital/I-Shou University, Kaohsiung 82445, Taiwan; shihwei8888@gmail.com; 7 Department of Obstetrics & Gynecology, E-Da Hospital/I-Shou University, Kaohsiung 82445, Taiwan

**Keywords:** *Bulnesia sarmientoi*, supercritical fluid extraction, human lung cancer cells, necroptosis, migration and invasion, chemical ingredient analysis

## Abstract

*Bulnesia sarmientoi* (BS) has long been used as an analgesic, wound-healing and anti-inflammatory medicinal plant. The aqueous extract of its bark has been demonstrated to have anti-cancer activity. This study investigated the anti-proliferative and anti-metastatic effects of BS supercritical fluid extract (BSE) on the A549 and H661 lung cancer cell lines. The cytotoxicity on cancer cells was assessed by an MTT assay. After 72 h treatment of A549 and H661 cells, the IC_50_ values were 18.1 and 24.7 μg/mL, respectively. The cytotoxicity on MRC-5 normal cells was relatively lower (IC_50_ = 61.1 μg/mL). BSE arrested lung cancer cells at the S and G_2_/M growth phase. Necrosis of A549 and H661 cells was detected by flow cytometry with Annexin V-FITC/PI double staining. Moreover, the cytotoxic effect of BSE on cancer cells was significantly reverted by Nec-1 pretreatment, and BSE induced TNF-α and RIP-1 expression in the absence of caspase-8 activity. These evidences further support that BSE exhibited necroptotic effects on lung cancer cells. By wound healing and Boyden chamber assays, the inhibitory effects of BSE on the migration and invasion of lung cancer cells were elucidated. Furthermore, the chemical composition of BSE was examined by gas chromatography-mass analysis where ten constituents of BSE were identified. α-Guaiene, (−)-guaiol and β-caryophyllene are responsible for most of the cytotoxic activity of BSE against these two cancer cell lines. Since BSE possesses significant cytotoxicity and anti-metastatic activity on A549 and H661 cells, it may serve as a potential target for the treatment of lung cancer.

## 1. Introduction

Lung cancer is one of the leading causes of death in the world, responsible for more than 1.5 million deaths worldwide annually [1]. There are two major subtypes of lung cancer, non-small-cell lung cancer (NSCLC) and small-cell lung cancer (SCLC). NSCLC accounts for approximately 80–85% of lung cancer cases. The high mortality rate of this disease may be related to the obscurity of the symptoms and its high metastasis rate. It is estimated around 57% of all lung cancers are metastatic in nature and can spread to various tissues and organs [2,3]. Various methods, such as chemotherapy, surgery, and radiation therapy, have been used to treat lung cancer patients. However, more effective therapies still need to be developed for curing the cancer, especially the treatment for the advanced stages of cancer.

Traditionally, two types of cell death under drug treatment are generally recognized: apoptosis and necrosis. Apoptosis is regarded as one form of programmed cell death, whereas necrosis is regarded as an uncontrolled and unregulated type of cell death. Recently, accumulating evidences have shown that necrosis can also be induced and proceed in a regular manner, which is a caspase-independent form of regulated cell death. This type of necrosis is called “programmed necrosis” or “necroptosis” [4]. For the necroptosis, upon stimulation of Fas/TNF receptor family to trigger extrinsic apoptotic signal, cells exhibit the morphology like necrotic cell death in the absence of caspase activity, and then could transduce necroptosis signal. In addition, the death of these necrotic cells was regulated by unique signaling pathway, dependent on receptor-interacting protein kinase 1 (RIP-1) and/or RIP-3 and specifically inhibited by the small molecule, necrostatin-1 (Nec-1) [5]. Necroptosis was found to associate with pathogenic effect on ischemic brain injury, myocardial infarction and neurodegeneration [6,7,8]. Drug resistance contributes to the high mortality of cancer, i.e., cancer cells can escape from apoptosis caused by chemotherapeutic drugs and this makes them resistant to chemotherapy. Several studies have revealed that necroptosis would accelerate the death of cancer cells by increasing the sensitivity of tumor cell to chemotherapy drugs [9,10,11]. Therefore, necroptosis provides an alternative process to enhance the sensitivity of cancer cells to chemotherapeutic agents, especially for the cases where the cancer cells are resistant to apoptosis. These properties make necroptosis a new target to eliminate cancer cells when caspases are inhibited or defective [12,13].

Metastasis is a critical step in cancer progression and is the leading cause of cancer-related deaths in human. Tumor metastasis is a complex multistep process, including tumor angiogenesis, cell migration, cell attachment, degradation of extracellular matrix (ECM), invasion and proliferation. Through these events, cancer cells spread primarily through the thin wall of the ECM into the blood vessels or lymphatic circulation system to colonize distant organs, and ultimately resulting in distant metastasis [14,15,16].

Some plants were reported to have anti-lung cancer effects, including *Podophyllum peltatum*, *Taxus brevifolia* Nutt., *Catharanthus roseus*, and *Camptotheca acuminata*. Some of their bioactive compounds had been developed as chemotherapeutic drugs, such as podophyllotoxin derivatives [17,18], taxol [19], vinca alkaloids [20] and camptothecins [21]. Most of them exhibit apoptotic effects on tumors, while some agents trigger necroptosis in cancer cells [22,23,24,25,26]. Shikonin was the first compound that was reported to have the ability to induce necroptosis [27]. Subsequently, shikonin and its analogs were reported successively to have high cytotoxicity on cancer cells, as well as the ability to avoid drug resistance [28,29,30,31]. Furthermore, shikonin could reduce the lung metastasis of osteosarcoma by inducing necroptosis [29].

Supercritical fluid extraction (SFE), generally performed by carbon dioxide (CO_2_), has been widely recognized as a green sample preparation technique and has several advantages of non-toxicity, efficiency, high separation selectivity and environmental friendliness, while it avoids thermal degradation and can be recyclable. Especially, due to the lipophilic nature of CO_2_, SFE is very suitable for the extraction of non-polar or moderately polar ingredients. Therefore, the use of SFE to extract plants is rapidly increasing [32,33,34].

*Bulnesia sarmientoi* (BS, Palo Santo), an endemic tree in the Gran Chaco area around Argentina, Bolivia, Brazil, and Paraguay borders, belongs to the Zygophyllaceae family, which is frequently used to produce wood furniture, handicrafts, Buddha tables, and flooring. The wood waste of BS is often used to extract essential oils, which have the balmy rose or violet aroma, and have been used in perfumery and aromatherapy [35]. Besides this, BS has been used as a traditional medicine in analgesic, wound healing, anti-inflammation, antioxidant, bactericidal activities, to improve serum lipid profiles and treat gastrointestinal problems [35,36]. Aqueous extract of BS (aqBSE) exhibited anti-platelet activity and thrombus formation via MAP kinase inhibition [37]. BS has also shown anti-tumor activity. The aqBSE could induce apoptosis of A549 lung cancer cells via p53 induction and decrease the tumor size in subcutaneous sarcoma 180 tumor-bearing nude mice [38]. A similar apoptotic effect of aqBSE on lung cancer H460 cells was also reported [39]. A further study demonstrated that (−)-epicatechin isolated from aqBSE could enhance the apoptosis of SW480 human colon cancer cells by Bax and p53 induction and Bcl-2 down-regulation [40]. Instead of the aqueous extract, this study evaluates the anti-cancer potential of BS SFE extract (BSE) on lung cancer cells. The inhibitory effects of BSE on cell proliferation, migration and invasion of lung cancer A549 and H661 cells were investigated. Furthermore, the cell necroptosis induced by BSE was also elucidated.

## 2. Results and Discussion

### 2.1. Effects of BSE on Anti-Proliferation of Human Lung Cancer Cells

**Figure 1:**
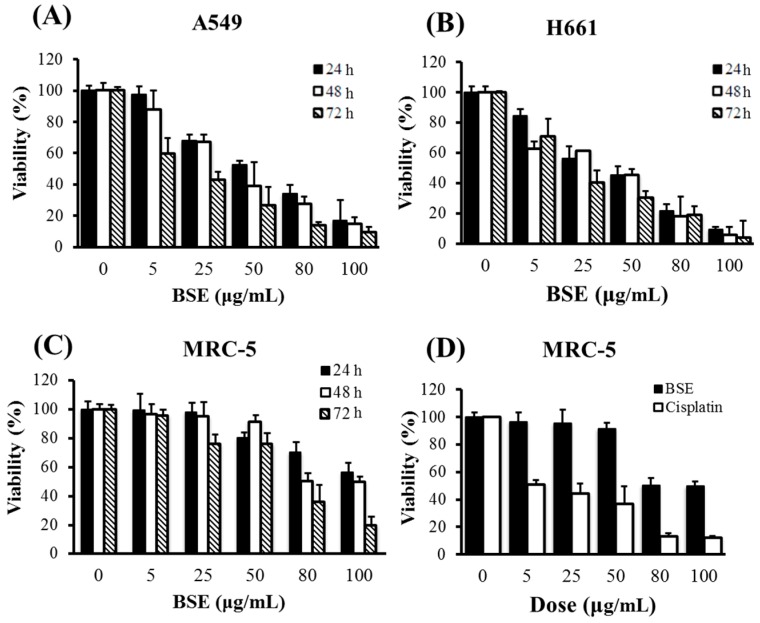
Effects of treatment concentration and duration of BSE on the proliferation of (**A**) lung cancer A549 cells, (**B**) H661 cells, (**C**) lung fibroblast MRC-5 normal cells, (**D**) the comparison of the effects of BSE and cisplatin on MRC-5 cells under 48 h treatment.

The cytotoxicities of BSE on A549 and H661 human lung cancer cells and human fetal lung fibroblast MRC-5 normal cells are shown in Figure 1. The treatments were performed at different doses for 24, 48 and 72 h, respectively. From the data shown in the figure, BSE exhibited the cytotoxicities on each of these three cell lines in a dose-dependent manner. On the other hand, Table 1 shows that the longer the treatment time, the greater the cytotoxicity. Among these three cell lines, BSE exhibited a much lower toxicity to MRC-5 normal cells. When comparing to the clinical anti-cancer drug cisplatin, BSE and cisplatin had similar cytotoxicity on lung cancer cells, but BSE appeared less toxic to MRC-5 normal cells than cisplatin. It is worth noting that cisplatin had higher toxicity to the normal lung cells than the lung cancer cells.

Mollah et al. [38,39] reported that the IC_50_ values of aqBSE on A549 and H460 cells for 24 h treatment were around 225 μg/mL and 75 μg/mL, respectively. In comparison, the cytotoxicity of BSE is higher, i.e., the IC_50_ values for 24 h treatment were 59.0 μg/mL and 46.6 μg/mL on A549 and H661 cells, respectively. As mentioned previously, SFE is suitable for extracting non-polar or moderately polar ingredients; while the aqueous extraction would get the polar constituents of BS. Thus, the deviation of the cytotoxicities of these two extracts might be due to the difference of their compositions.

### 2.2. Effects of BSE on Cell Cycle Regulation of Lung Cancer Cells

**Figure 2:**
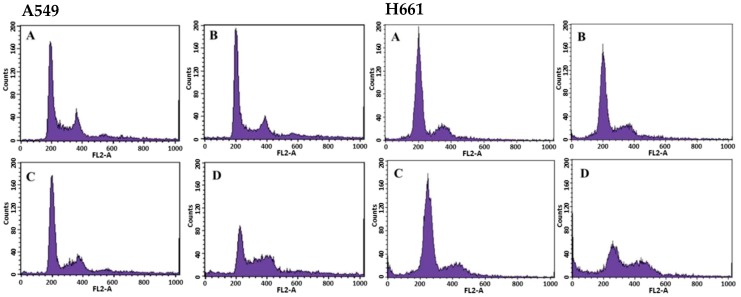
Cell cycle analysis of A549 and H661 cells treated with different concentrations of BSE by flow cytometry. The cells (1 × 10^6^ cells) were incubated with: (**A**) 0 μg/mL (Control), (**B**) 22 μg/mL, (**C**) 44 μg/mL, (**D**) 88 μg/mL of BSE for 48 h. The percentage of each phase was analyzed by WinMDI 2.5 software.

The effects of BSE on cell cycle progression in A549 and H661 cells were investigated by the quantitation of the cell cycle distribution under treatment with different BSE concentrations for 48 h by flow cytometry (Figure 2). For these two cell lines, the number of cells in the S phase and the G_2_/M phase rose significantly with increasing the BSE concentration from 0 to 88 μg/mL (Table 2). This experimental finding implies that BSE could arrest A549 and H661 cells at S and G_2_/M phase. In contrast, the aqBSE arrests A549, S180 and H460 lung cancer cells at the sub-G1 phase [38,39].

### 2.3. BSE Treatment Caused A549 and H661 Necrosis but not Apoptosis

**Figure 3:**
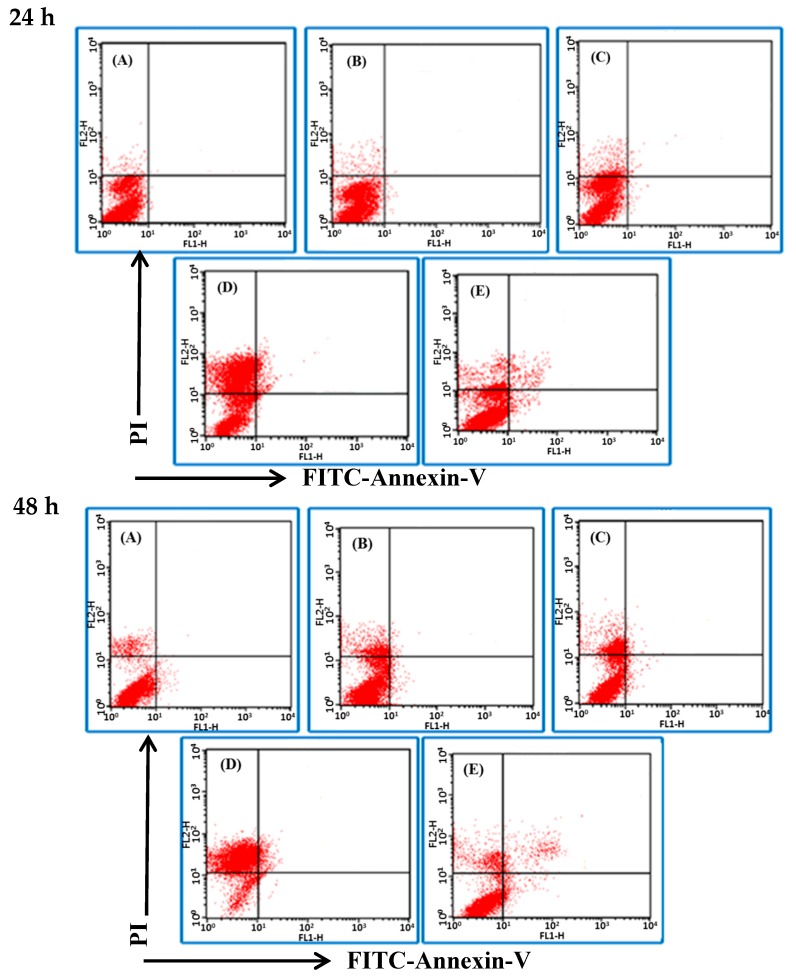
Effects of BSE on H661 cell death pattern under the treatment of different durations (24 and 48 h) and different concentrations of BSE: (**A**) 0 μg/mL (Control), (**B**) 22 μg/mL, (**C**) 44 μg/mL, (**D**) 88 μg/mL, (**E**) Cisplatin in 100 μg/mL. Lower left (LL) quadrant: viable cells; upper left (UL) quadrant: necrotic cells; lower right (LR) quadrant: early apoptotic cells; upper right (UR) quadrant: late apoptotic cells.

The Annexin V-FITC/PI detection kit was used to stain the cells and to examine the influence of BSE on cancer cell death by flow cytometry. Binding of Annexin V to phosphatidylserine (PS) of cells and the staining of PI on nucleus are the unique properties of apoptotic cell and necrotic cell, respectively. The analytical results are expressed in four quadrants (Figure 3). In general, the dots in the lower left (LL, annexin V^−^/PI^−^) quadrant are viable cells; those in the upper left (UL, annexin V^−^/PI^+^) quadrant are cells in necrosis; while those in the lower right (LR, annexin V^+^/PI^−^) quadrant are cells undergoing early apoptosis; upper right (UR, annexin V^+^/PI^+^) quadrant are cells in late apoptosis. Figure 3 shows the cell death pattern of H661 cancer cells under the BSE treatment. The dots dispersed and shifted to the UL side, i.e., in necrosis, in a dose- and time-dependent manner.

**Figure 4:**
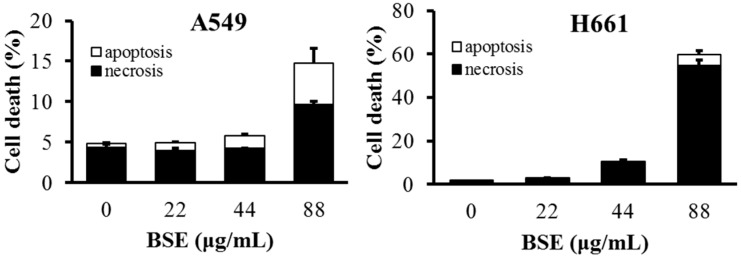
The ratio of apoptosis and necrosis induced by different concentrations of BSE on A549 and H661 cells for 24 h treatment.

The flow data show that BSE had no significant effect on the induction of apoptosis on H661 cells. In contrast, cisplatin induced some cells to die in necrosis, while some died by apoptosis (Figure 3E). The same analysis was also performed on A549 cells and a similar necrotic effect was observed (Figure 4). These data demonstrate that the major type of cell death of H661 and A549 stimulated by BSE is necrosis but not apoptosis.

### 2.4. Effect of BSE on Protein Expression Related to Necroptosis

**Figure 5:**
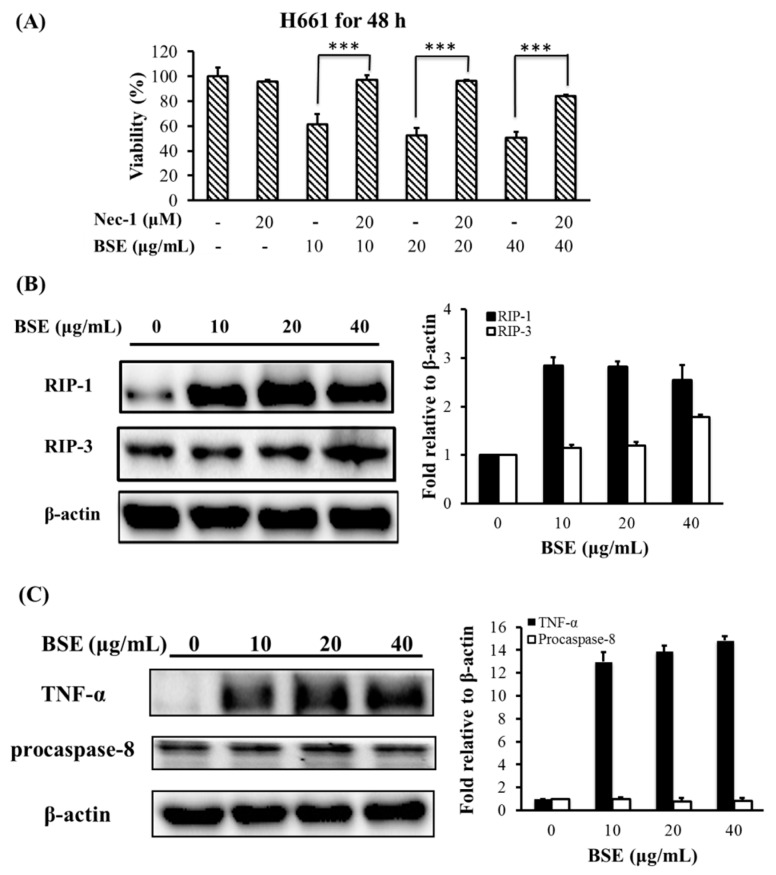
BSE induces cell death via necroptotic pathway in the H661 cells. (**A**) Cytotoxic effect of BSE is reverted by Nec-1. H661 cells were pretreated with or without 20 μM nec-1 for 30 min and then treated with different doses of BSE (10, 20 and 40 μg/mL) or DMSO (0 μg/mL) for 48 h. The viability of these cells was measured by MTT assay. A significant difference between the denoted two experiments was indicated as *** *p* < 0.001. (**B**) BSE induces RIP-1 expression in H661 cells; (**C**) BSE induces TNF-α expression in the absence of caspase-8 activity in H661 cells. Cell extracts from BSE administration were harvested at 24 h and subjected to western blot analysis. Densitometric analyses of protein were normalized to the loading control β-actin.

As mentioned earlier, necroptosis is a form of regulated cell death. The expression of RIP-1 and/or RIP-3, and specifically inhibited by Nec-1 are the crucial characteristics of necroptosis [4,5,41,42]. To characterize the type of necrosis that BSE triggered, we first tested whether Nec-1 could inhibit BSE-stimulated necrosis in lung cancer cells. The H661 cells were pretreated by 20 μM Nec-1 for 30 min, and then treated with BSE. Data in Figure 5A show that Nec-1 nearly completely inhibited BSE-induced cell death in H661 cells. This finding suggests that the cell death induced by BSE in H661 cells was necroptosis. In addition, Figure 5B shows that the RIP-1 was highly expressed on BSE-treated H661 cells, while the level of RIP-3 was only slightly enhanced under BSE treatment. These results indicate that the type of necrosis triggered by BSE was necroptosis through RIP-1 induction.

Necroptosis could be induced by stimulating death receptors with agonists such as TNF-α, FasL, and TRAIL [5,41]. TNF-α stimulation can transduce necroptosis signal in the absence of caspase-8 activity [43]. Figure 5C shows that TNF-α was highly expressed when H661 cells were treated with 10 to 40 μg/mL of BSE. Moreover, the protein level of procaspase-8 had no significant change under BSE treatment. Accordingly, these results indicate that the necroptosis might be stimulated by TNF-α in the absence of caspase-8 activity.

In contrast, Mollah et al. [38,39] demonstrated that aqBSE triggers lung cancer cell death through the apoptosis process by the evidences of DNA fragmentation, annexin V staining, and the results of immunoblot analysis, i.e., treatment with aqBSE would increase the expressions of p53 and Bax and down-regulate Bcl-2 protein in cancer cells.

### 2.5. BSE Inhibits the Migration and Invasion of A549 and H661 Cells

**Figure 6:**
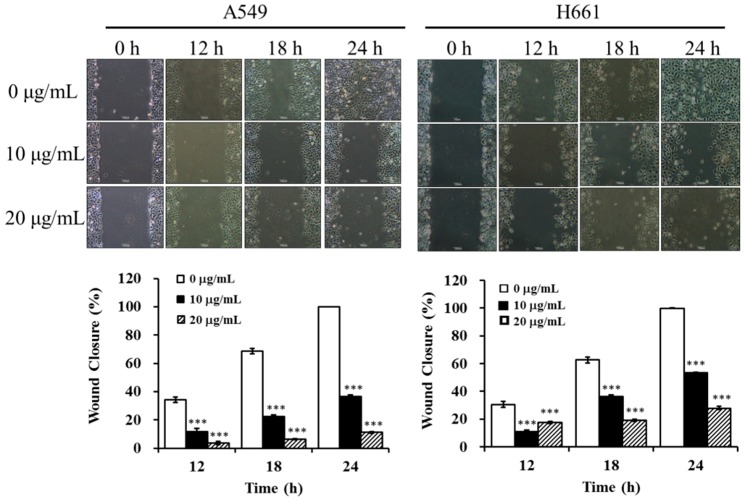
Effect of BSE on A549 and H661 cell migration by wound healing assay. A549 and H661 were seeded into culture dishes and grown to 90% confluence in 6 cm dishes. The cells were scratched with a sterile tip, BSE (0, 10 and 20 μg/mL) were added and the wounded area were photographed at 0, 12, 18 and 24 h. The inhibitory effect on cell migration is expressed as the level of wound closure. A significant difference from the vehicle was indicated as *** *p* < 0.001 (Student’s *t*-test).

**Figure 7:**
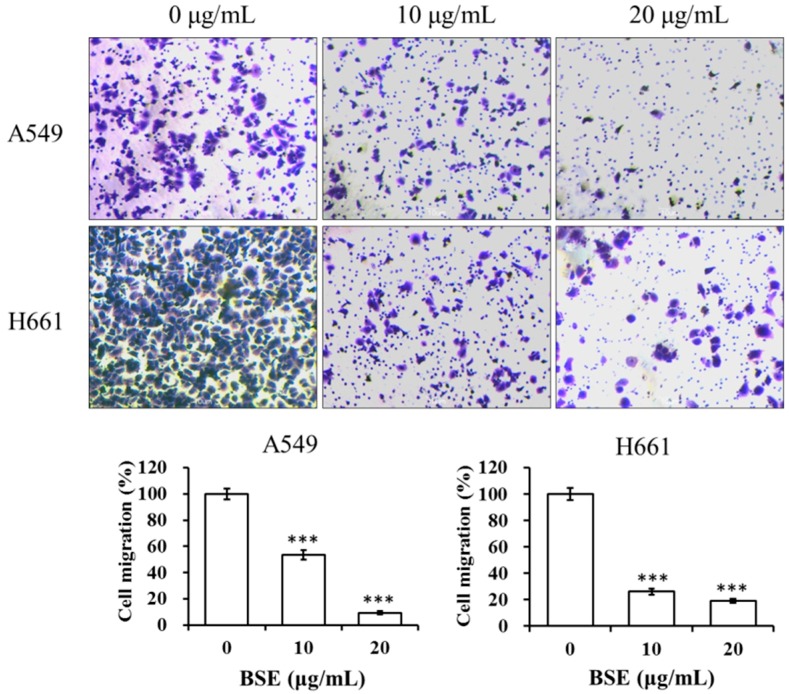
Effect of BSE on A549 and H661 cell migration by transwell assay. An aliquot of 2 × 10^5^ cells of A549 and H661, respectively, were seeded onto the upper chamber in serum-free medium with BSE (0, 10 and 20 μg/mL) for 24 h. The cells migrated through polycarbonate membrane were stained with crystal violet and photographed. The crystal violet-stained cells were calculated. A significant difference from the vehicle was indicated as *** *p* < 0.001 (Student’s *t*-test).

To study the anti-metastatic effect of BSE on lung cancer cells, the inhibitory effects of BSE on migration and invasion of cancer cells were investigated by using the wound healing assay and Boyden chamber assay. At first, A549 and H661 cells were incubated in the absence or presence of BSE for the wound healing assay. Figure 6 shows that incubation with BSE significant decreased the migration of A549 and H661 cells into the wounded area in a time- and dose-dependent manner. Comparing these results with the control (0 μg/mL), the treatment at 10 and 20 μg/mL of BSE reduced the cancer cell migration by around 64% and 89% on A549 cells and by around 47% and 72% on H661 cells at 24 h, respectively. Furthermore, the results of Boyden chamber assay using a transwell chamber are demonstrated in Figure 7. The stained migratory cells treated by 10 and 20 μg/mL BSE were significantly reduced by around 46% and 90% comparing with the control on A549 cells and around 74% and 81% on H661 cells.

**Figure 8:**
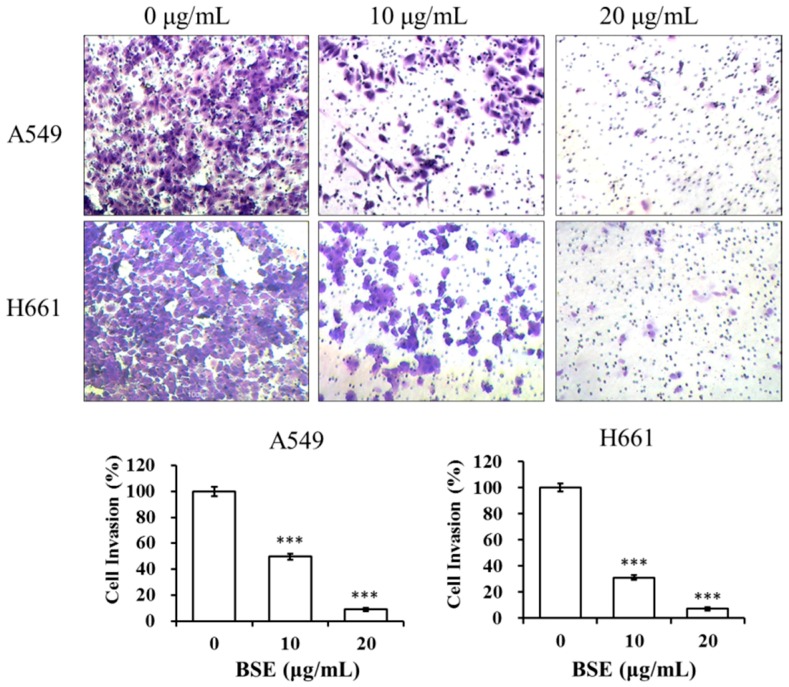
Effect of BSE on A549 and H661 cell invasion. A BD BioCoat^TM^ Matrigel^TM^ invasion chamber was used for invasion assay. An aliquot of 2 × 10^5^ cells of A549 and H661, respectively, were seeded onto the upper chamber in serum-free medium with BSE (0, 10 and 20 μg/mL) for 24 h. The cells invaded through Matrigel were stained with crystal violet and photographed. The crystal violet-stained cells were calculated. A significant difference from the vehicle was indicated *** *p* < 0.001 (Student’s *t*-test).

The inhibitory effects of BSE on the invasion of lung cancer cells were examined by a cell invasion assay with the Boyden chamber coated with collagen. The number of A549 and H661 cells that invaded through the Matrigel was significantly inhibited by BSE treatment (Figure 8). Quantification of cells in the lower chamber indicates that BSE treatment significantly inhibited A549 and H661 cell invasion in a concentration-dependent manner. The cell invasion rate is expressed as a percentage to the control (0 μg/mL). When treating with 10 μg/mL BSE, the percentage of invasive cells decreased to 50% and 31% on A549 and H661 cells, respectively. While at the concentration of 20 μg/mL BSE, the cell invasions were almost totally inhibited, i.e., the invasive activity were 9% and 7% on A549 and H661 cells, respectively. In summary, these results suggested that BSE displayed strong inhibitory effects on the migration and invasion of A549 and H661 cancer cells.

### 2.6. Cytotoxicity of Main Ingredients of BSE on Lung Cancer Cells

**Figure 9:**
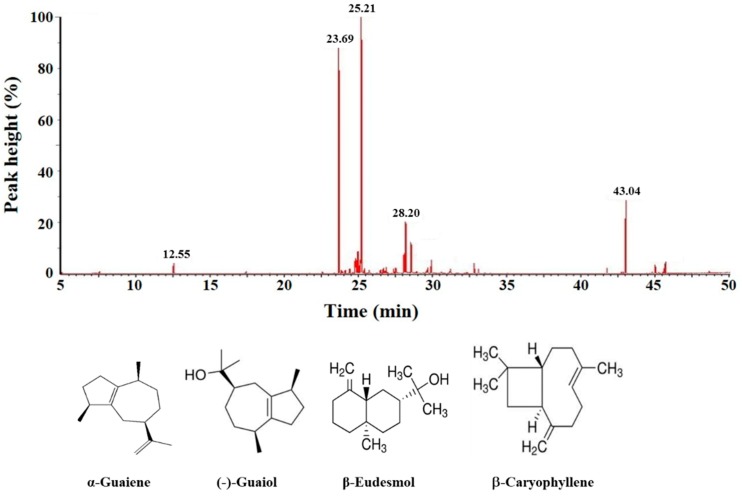
Gas chromatography-mass spectrometry profile of BSE sample and the four ingredients identified from this analysis. These four compounds were confirmed by comparing with their mass spectral data with the NIST mass spectral library and commercially available products.

The gas chromatography-mass spectrometry (GC-MS) analytical results show that BSE contains at least 25 ingredients (Figure 9), of which 10 compounds were identified using the NIST spectral database of the mass spectrometry. The main constituents of BSE were identified as α-guaiene (relative content RC = 34.0%; retention time RT = 25.21 min), (−)-guaiol (RC = 28.7%; RT = 23.69 min), (−)-nortrachelogenin (RC = 8.3%; RT = 43.04 min), β-eudesmol (RC = 6.4%; RT = 28.20 min) and β-caryophyllene (RC = 1.4%; RT = 12.55 min). The identities of α-guaiene, (−)-guaiol, β-eudesmol and β-caryophyllene were further confirmed by comparing their mass spectral data with the analytical results from the commercially available products, while (−)-nortrachelogenin was commercially unavailable.

**Figure 10:**
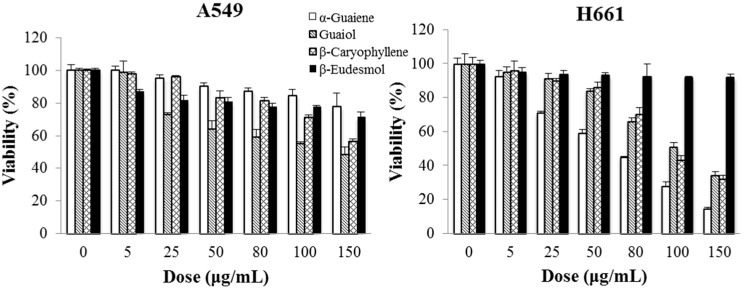
Cytotoxicities of α-guaiene, (−)-guaiol, β-caryophyllene and β-eudesmol on A549 and H661 cells for 48 h treatment.

The cytotoxicity assessments of these four compounds on two lung cancer cell lines for 48 h treatment are shown in Figure 10. The cytotoxicity (expressed by IC_50_ value) of α-guaiene, (−)-guaiol and β-caryophyllene to A549 cells were 142.6 ± 5.1 μg/mL (0.698 ± 0.025 mM), 110.8 ± 4.0 μg/mL (0.618 ± 0.022 mM) and 150.2 ± 3.7 μg/mL (0.685 ± 0.017 mM), respectively. To H661 cells, the IC_50_ values of these three ingredients were 69.9 ± 1.3 μg/mL (0.342 ± 0.006 mM), 111.9 ± 3.1 μg/mL (0.624 ± 0.017 mM) and 106.2 ± 3.3 μg/mL (0.520 ± 0.016 mM), respectively. The cytotoxicities of β-eudesmol to these two cancer cell lines were insignificant. Therefore, α-guaiene, (−)-guaiol and β-caryophyllene should be responsible for the main cytotoxicity of BSE against A549 and H661 cells.

α-Guaiene is a sesquiterpene and exists in the extracts of numerous plants [44,45,46]. However, there has been little literature reported on its anti-cancer activity. (−)-Guaiol is a sesquiterpene alcohol and is a key component of many medicinal plants [47,48]. It has been demonstrated that (−)-guaiol possesses significant cytotoxicity on NSCLC cells and regulates autophagic cell death [49,50]. β-Caryophyllene, an important component in the extracts of various species of medicinal plants, has been reported to have anti-proliferative effects on various cancer cells, including pancreatic cancer, colon cancer [51], hepatoma [52], endometrial cancer [53], and skin epidermoid cancer [54].

Rodilla et al. [35] reported that the major components of their BS essential oil were bulnesol (RC = 34.7%) and guaiol (RC = 20.4%). α-Guaiene (RC = 0.2%), β-caryophyllene (RC = 0.1%) and β-eudesmol (RC = 1.3%) were also presented but in a small amount. The difference in the composition between this essential oil extract and BSE should be mainly due to the difference in extraction methods, in which the former and the latter were prepared by steam distillation and SFE, respectively.

As for the aqBSE, the analytical results from high performance liquid chromatography (HPLC) show that the main active ingredients are various types of catechins, such as (−)-epigallocatechin, (−)-epicatechin, (−)-epicatechin gallate, and (+)-catechin gallate; and these catechins have their own anti-cancer effects [39].

## 3. Materials and Methods

### 3.1. Reagents

Dulbecco’s modified Eagle’s medium (DMEM), fetal bovine serum, RPMI-1640 medium, l-glutamine, penicillin and streptomycin were from Gibco (Grand Island, NY, USA). 3-(4,5-Dimethylthiazol-2-yl)-2,5-diphenyl tetrazolium bromide (MTT) and necrostatin-1 reagent were purchased from Sigma-Aldrich (St. Louis, MO, USA). Antibodies to RIP-1 and TNF-α were purchased from Cell Signaling Technology (Danvers, MA, USA). Primary antibody to RIP-3 and the horseradish peroxidase-conjugated anti-rabbit and anti-mouse IgG secondary antibodies were obtained from Millipore Co. (Temecula, CA, USA). Antibodies to procaspase-8 and β-actin were separately purchased from BD Bioscience (San Jose, CA, USA) and Santa Cruz Biotechnology (Santa Cruz, CA, USA). All chemicals used in this study were of reagent or higher grade.

### 3.2. Preparation of BSE

The wood waste material of BS (imported from South America) was purchased from a local wood furniture company (Chiayi, Taiwan). The supercritical-CO_2_ fluid extract (BSE) was prepared by processing 2 kg of the crushed powder in the supercritical fluid extractor (5 L/1000 bar R&D unit, Natex, Ternitz, Austria). The dynamic extractions were performed as: 0–150 bar for 20 min; 150–250 bar for 20 min; 250–300 bar for 10 min; 300–350 bar for 10 min; and stay at 350 bar, 40 °C for 2 h. The extracted sample was dried in a freeze-dryer. The resulting extract was 208 g. The extraction yield was 10.4%. The BSE was stored at −20 °C and dissolved in dimethyl sulfoxide (DMSO) before use. The final DMSO concentration in the medium was less than 0.1%.

### 3.3. Cell Culture

Human lung carcinoma cell line A-549 (BCRC 60074), H661 (BCRC 60125) and human lung normal fibrobast MRC-5 (BCRC 60023) were purchased from Bioresource Collection and Research Center (BCRC, Hsinchu, Taiwan). A-549 and MRC-5 cells were grown in DMEM with 10% fetal bovine serum, 1% penicillin/streptomycin, and supplemented with 2 mM l-glutamine. H661 cells were grown in RPMI-1640 medium supplemented with 10% fetal bovine serum, 1% penicillin/streptomycin, and 2 mM l-glutamine. Cells were subcultured every two days and cultivated in a humidified incubator at 37 °C with 5% CO_2_ and 95% air.

### 3.4. Cytotoxicity Assay

Cancer cells were cultured in 96-well plates at 1 × 10^4^ cells for 24 h before treatment. The indicated concentration of BSE was then added. After cultivation for the given period, the culture medium was replaced by 100 μL of the culture medium containing 0.5 mg/mL MTT assay kit (Sigma-Aldrich Chemicals), and the cells were further incubated for 2 h. The medium solution was removed and an aliquot of 100 μL DMSO was added. The plate was shaken until the crystals dissolved. The cytotoxicity against the tested cells was measured at 570 nm using an ELISA reader (Model 550, Bio-Rad Laboratories, Hercules, CA, USA).

### 3.5. Flow Cytometric Analysis on Cell Cycle

Lung cancer cells (1 × 10^6^ cells) were incubated in a 10 cm dish for 24 h. Treated with various concentrations of BSE for 48 h. The treated cells were harvested, washed with phosphate-buffer saline (PBS) and treated with 100 μL trypsin-EDTA solution to detach the cells. The suspension solution was collected and centrifuged at 200× *g* for 10 min. The cell pellet was washed with PBS, and then fixed in 70% ethanol at 4 °C overnight. After washing twice with cold PBS, cells were suspended in PBS that contained 40 μg/mL propidium iodide (PI) and 0.1 mg/mL RNase A (Gentra Systems Inc., Minneapolis, MN, USA), and was kept at 4 °C for 12 h. The stained cells were then analyzed with BD FACSCalibur^TM^ flow cytometer (BD Bioscience, Franklin Lakes, NJ, USA). The percentages of cell cycles were calculated by WinMDI 2.9 software (TSRI, La Jolla, CA, USA).

### 3.6. Measurement of Apoptotic Ratio of A549 Cells

The necrotic and apoptotic cells were analyzed with Annexin-V-FITC apoptosis detection kit (AVK050, Strong Biotech Co., Taipei, Taiwan). Lung cancer cells (1 × 10^6^ cells) were incubated in 10 cm dish for 24 h. After treating with different concentrations of BSE for 24 and 48 h, the treated cells were harvested, washed with PBS, and then treated with trypsin-EDTA solution to detach the cells. The suspended cells were centrifuged at 200× *g* for 10 min. After washing twice with cold PBS, cells were collected by centrifugation at 200× *g* for 5 min, and stained with FITC-conjugated annexin V and propidium iodide (PI). Cytometry was performed on BD FACSCalibur^TM^ flow cytometer and analyzed with WinMDI 2.9 software.

### 3.7. Cell Migration by Wound Healing Assay

An aliquot of 1 × 10^6^ cells were seeded into 6 cm dish and cultured for 24 h. Cells wound was artificially injured by using a sterile pipette tip. The cells were washed twice with PBS buffer to remove detached cells and cell debris. After which the culture medium containing certain dose of BSE was added. Cell migration into the wounded area was photographed at different time points using an inverted phase-contrast microscope (Nikon Eclipse TS100, Chiyoda-ku, Tokyo, Japan) at a magnification of 4×. All wound healing assays were conducted in triplicate. The closure rate was calculated using the following formula:

Wound closure rate (%) = (Original width − Width after migration)/Original width × 100%

### 3.8. Cell Migration and Invasion Assays by Boyden Chamber

Cell migration and invasion were evaluated with the CytoSelect™ 24-well cell migration assay (Cell Biolabs, San Diego, CA, USA) and BioCoat™ Matrigel™ Invasion Chamber (BD Biosciences), respectively. To determine the effect of BSE on cell migration, 2 × 10^5^ lung cancer cells with 0, 10 and 20 μg/mL BSE in 200 μL serum-free medium were placed on a polycarbonate membrane insert (8 μm pore size), and then put this insert on the bottom chamber which was filled with DMEM or RPMI-1640 medium containing 10% FBS as a chemo-attractant. After incubation at 37 °C for 24 h, the non-migratory cells in the upper chamber were wiped away by scrubbing with wet cotton-tipped swabs for 2–3 times. The invaded cells were fixed with methanol, stained with crystal violet, photographed and counted under a phase-contrast microscope (400×, Nikon Eclipse TS100, Chiyoda-ku, Tokyo, Japan). The similar procedure was carried out in experiments on cell invasion, except that the polycarbonate membrane was coated with a uniform layer of collagen.

### 3.9. Western Blot Analysis

To analyze the related proteins, 1.5 × 10^6^ cells were seeded into 10 cm culture dish with or without BSE treatment. After 24 h incubation, the medium was removed and cells were washed several times with PBS (0.01 M, pH 7.2). Whole-cell lysates were prepared as our previous paper [53]. The harvested protein concentration was measured using a protein assay kit (Bio-Rad). Samples with equal amounts of denatured proteins were resolved on 10% sodium dodecyl sulfate polyacrylamide gel electrophoresis (SDS-PAGE). Proteins were transferred onto a nitrocellulose membrane (Immunobilin P; Millipore, Billerica, MA, USA). The membrane was blocked with 5% nonfat milk in Tris-buffered saline, probed with indicated primary antibody and then hybridized with horseradish peroxidase-conjugated secondary antibodies. The protein levels were detected using Enhanced Chemiluminescence (ECL) Plus Western blotting detection reagents (Amersham Bioscience, Uppsala, Sweden). Densitometric analyses were conducted by ChemiDoc^TM^ XRS+ System (Bio-Rad).

### 3.10. Gas Chromatography-Mass Spectrometry

GC-MS analysis was performed using Clarus 500 GC and Clarus 500 system (Perkin Elmer, San Diego, CA, USA) with the electron impact mode (70 eV) injector. The GC column was ZB-5 capillary column (30 m × 0.25 mm, film thickness 0.1 μm, FactorFour^TM^, Phenomenex, Torrance, CA, USA). Injector temperature was set at 150 °C. Oven temperature was kept at 55 °C for 5 min, then raised to 300 °C by a rate of 5 °C/min, and kept for 10 min. The carrier gas was helium at a flow rate of 1 mL/min. Diluted samples of 1.0 μL were injected manually and the split ratio was 1:5. The percentages of the compounds were calculated by the area normalization method without considering response factors. The components were identified by comparison of their mass spectra with the NIST MS 2.0 database (NIST, Gaithersburg, MD, USA). Four authentic compounds used to confirm the MS-identified ingredients and used in the cytotoxicity experiment on lung cancer cells were purchased from Sigma-Aldrich.

### 3.11. Statistical Analysis

All experiments were carried out for three to five independent replicates. The experimental data were analyzed by Microsoft Excel software (2010, Microsoft Software Inc., Redmond, WA, USA). The data are expressed in terms of mean and standard deviation. The statistical differences were analyzed by Student’s *t*-test.

## 4. Conclusions

This study demonstrates that the supercritical fluid extract of BS (BSE) had significant cytotoxicity on A549 and H661 lung cancer cells. Under BSE treatment, the cell cycle arrest, necrosis by annexin V-FITC/PI double staining detection, and expressions of necroptotic proteins in cancer cells were investigated. These results indicate that BSE exhibited necroptotic effect on NSCLC cells. Additionally, BSE had significant suppressive effects on the migration and invasion of lung cancer cells. The experimental data of chemical composition analysis and the bioactivity assay indicate that α-guaiene, (−)-guaiol and β-caryophyllene were mostly responsible for the anti-cancer activity of BSE. In comparison to the aforementioned literature of the aqBSE, the main ingredients of BSE were quite different from those of aqBSE. This might result in the different cell death types (necroptosis vs apoptosis) and their cytotoxicities. Since BSE is prepared by SFE, which is an environment-friendly and efficient technique, as well as possesses a high cytotoxicity toward NSCLC cells, BSE represents a potential source for the treatment of lung cancer.

## Figures and Tables

**Table 1:** Cytotoxicities (expressed by IC_50_ value) of BSE and cisplatin on different lung cells.

Cell Line	BSE (μg/mL)			Cisplatin (μg/mL)		
	24 h	48 h	72 h	24 h	48 h	72 h
A549	59.0	43.7	18.1	58.7	44.3	19.2
H661	46.6	44.6	24.7	79.6	16.9	16.1
MRC-5	120.8	89.7	61.1	34.1	10.7	7.8

**Table 2:** Distribution of lung cancer cells arrested at different phases after the treatment of different BSE concentrations for 48 h *.

BSE Conc. (μg/mL)	A549 Cells				H661 Cells			
	SubG_1_ (%)	G_0_/G_1_ (%) *	S (%) *	G_2_/M (%) *	SubG_1_ (%)	G_0_/G_1_ (%) *	S (%) *	G_2_/M (%) *
0	0.37 ± 0.08	65.89 ± 1.33	10.61 ± 0.55	23.50 ± 1.83	2.96 ± 0.40	76.75 ± 0.51	4.72 ± 0.29	18.53 ± 0.23
22	0.56 ± 0.07	65.87 ± 3.50	9.99 ± 0.58	24.15 ± 3.30	4.48 ± 0.50	77.64 ± 0.36	4.80 ± 0.33	17.56 ± 0.42
44	1.54 ± 0.10	65.06 ± 1.64	11.78 ± 0.49.	23.16 ± 2.12	5.97 ± 0.47	72.28 ± 0.99	7.33 ± 0.24	20.39 ± 1.04
88	4.59 ± 0.16	42.25 ± 2.10	19.86 ± 3.83	37.89 ± 5.93	14.17 ± 0.31	52.85 ± 2.33	11.43 ± 1.49	35.72 ± 2.88

* Percentages of the cell distribution of G_0_/G_1_, S and G_2_/M phases were calculated without the ratio of Sub-G1. The data are expressed as Mean ± SD in triplicate analysis.
